# Reduction-Triggered Breakable Micelles of Amphiphilic Polyamide Amine-*g*-Polyethylene Glycol for Methotrexate Delivery

**DOI:** 10.1155/2014/904634

**Published:** 2014-04-13

**Authors:** Yihang Huang, Jun Liu, Yani Cui, Huanan Li, Yong Sun, Yujiang Fan, Xingdong Zhang

**Affiliations:** National Engineering Research Center for Biomaterials, Sichuan University, 29 Wangjiang Road, Chengdu 610064, China

## Abstract

Reduction-triggered breakable polymeric micelles incorporated with MTX were prepared using amphiphilic PAA-*g*-PEG copolymers having S–S bonds in the backbone. The micelles were spherical with diameters less than 70 nm. The micelles could encapsulate the hydrophobic MTX in the hydrophobic core. The drug loading content and drug loading efficiency of the micelles were highly dependent on the copolymer chemical structure, ranging from 2.9 to 7.5% and 31.9 to 82.5%, respectively. Both the drug loading content and drug loading efficiency increased along with more hydrophobic segments in the copolymers. In normal circumstance, these micelles were capable of keeping stable and hold most of the MTX in the core, stabilizing the incorporated MTX through the *π*-*π* stacking with the phenyl groups in the backbone of the copolymers. In reductive environments that mimicked the intracellular compartments, the entire MTX payload could be quickly released due to the reduction-triggered breakage of the micelles. These micelles showed good antiproliferative activity against several cancer cell lines, including KB, 4T-1 and HepG2, especially within the low drug concentration scope.

## 1. Introduction

Chemotherapy is one of the major approaches for cancer treatment. Methotrexate (MTX) is a folate antimetabolite that blocks the synthesis pathway of DNA by inhabiting the activity of dihydrofolate reductase (DHFR) [1, 2]. It shows a greater toxic effect on rapidly dividing cancerous cells, which replicate their DNA much faster, than on normal cells. Thus, it is widely used in treatment of a number of human cancers [3]. However, the therapeutic effect of MTX is hindered by its toxic dose-related side effects, as well as the drug resistance by target cells. These drawbacks of MTX are closely related to its poor water-solubility and very short circulation half-life, which results in an essentially uniform tissue distribution [4]. Therefore, there is an urgent demand for formulations that are capable of efficiently enhancing drug targeting and reducing side effects [5].

In the past decades, many studies have demonstrated that nanoscaled drug delivery systems could encapsulate cytotoxic and poorly water soluble drugs for improving pharmacokinetic behavior, reducing toxicity, overcoming drug-resistance mechanisms, and enhancing tumor targeting through the enhanced permeation and retention (EPR) effect [6–8]. Polymeric micelles, formed from hydrophobic inner core and hydrophilic outer shell, have been demonstrated by many investigators to have potential usefulness in the process of tumor targeting drug delivery through intravenous injection [9–11]. The hydrophobic core functions as a nanoreservoir of hydrophobic drugs, whereas the hydrophilic outer shell improves solubility of hydrophobic drugs, provides a defense layer against attack of the reticuloendothelial system (RES), increases preservation of bioactive agents within the micellar core for long blood circulation, enhances targeting performance against tumor, and lessens the adverse effects of anticancer drugs [12, 13].

Advances in MTX delivery have been made by incorporating MTX with various nanoparticulate carrier systems, such as polymer-based particles [14], dendrimers [15–17], liposomes [18], micelles [19, 20], and inorganic nanoparticles [21]. In these rationally designed delivery carriers, intracellular translocation across the plasma membrane could be critical, because the nanoparticulate carriers generally show low cellular membrane permeability and enter the cell mainly through the endocytotic pathway with the formation of endosome [22]. It was reported that anticancer drugs delivered by nanoparticles were trapped in the endocytic vesicles, suggesting quite slow intracellular drug release due to the intracellular barriers of cellular organelle membrane [23–27].

Because the physicochemical properties and biological functions of tumor are different from that of normal tissues, drug carriers could be designed to release the loaded drugs after reaching the cancer cells by introducing sensitive functional groups in response to certain stimuli of the cancer cells [28]. Higher concentration of reductive glutathione (GSH) inside the tumor cells over normal cells provides a reducing intracellular environment as the inbuilt mechanism for release of anticancer drugs [29]. Therefore, controlling the release of anticancer drugs from polymeric micelles using reduction-sensitivity as a trigger to enhance tumor-killing efficacy and to minimize harmful side effects has been considered a promising way for intracellular drug delivery. Many researchers demonstrated that polymeric micelles, polymersomes, and nanogels containing S-S linkage prevented the premature release of loaded cargos in extracellular media but quickly released the DNA, siRNA, or drug cargoes inside the cells or under a reductive condition mimicking that of the intracellular compartments [30–34].

In our previous research [35, 36], amphiphilic reduction-triggered breakable micelles from polyamide amine-*g*-polyethyleneglycol (PAA-*g*-PEG) were developed to encapsulate the anticancer drug doxorubicin (DOX). In these micelles, phenyl groups were interspersed in the hydrophobic segment to interact with the aromatic structure of DOX through the *π*-*π* stacking, thus increasing the stability and drug loading ability. On the other hand, the hydrophilic segment of polyethyleneglycol (PEG) was oriented on the surface of the micelles, which determined the high biocompatibility of the micelles. In this work, 6 kinds of the PAA-*g*-PEG micelles with different hydrophobic/hydrophilic ratio were investigated as nanocarrier for MTX. MTX was encapsulated in PAA-*g*-PEG micelles using solvent dispersion/dialysis process. The size and morphology of drug-loaded micelles were characterized by DLS and TEM. Drug loading content, drug loading efficiency, and drug release behavior under reductive condition were studied using ultraviolet spectroscopy. The antiproliferative activity of MTX-incorporated micelles was measured against different tumor cells in comparison with those of free MTX.

## 2. Materials and Methods

### 2.1. Materials

Acryloyl chloride, phenethylamine, and ethanolamine were purchased from Sigma-Aldrich. Cystamine dihydrochloride, N,N′-dicyclohexyl carbodiimide (DCC), dimethylamine pyridine (DMAP), triethylamine (TEA), and succinic anhydride were purchased from Asta Tech Pharmaceutical Co., Ltd. (Chengdu, China). Polyethyleneglycol monomethyl ether (MPEG, Mn 2000 and 5000) were obtained from Fluka and were dried by azeotropic distillation from dry toluene immediately before used. Methotrexate (MTX) was purchased from Aladdin Reagent Inc. (Shanghai, China). All other agents are of analytical grade. The chemicals were used as-received unless otherwise addressed.

### 2.2. Synthesis of the Reduction-Degradable PAA*-g*-PEG Copolymers

Preparation of reduction-degradable PAA-*g*-PEG copolymers was performed by the method reported previously [35]. Briefly, cystamine bisacrylamide was synthesized by reacting acryloyl chloride with cystamine dihydrochloride in dichloromethane/water. Then, the freshly obtained cystamine bisacrylamide (1.044 g, 4 mmol) was reacted with a mixture of phenethylamine and ethanolamine (total 4 mmol, mol ratio: phenethylamine/ethanolamine = 8/2, 7/3, and 6/4, resp.) at 125°C under Ar atmosphere to acquire PAA containing disulfide linkage. Finally, as an example, amphiphilic PAA-*g*-PEG (PAA(8:2)-PEG2000) copolymers were obtained by coupling *α*-carboxy-*ω*-methoxy polyethyleneglycol (MPEGCOOH, 1.05 g, Mn = 2000, 0.5 mmol of carboxyl) on PAA (0.74 g, phenethylamine/ethanolamine = 8/2, 0.4 mmol of hydroxyl group) using DCC (0.124 g, 0.7 mmol) as coupling agent and DMAP (0.061 g, 0.05 mmol) as catalyst in dry DMSO at room temperature. Following this way, six kinds of amphipathic reduction-degradable PAA-*g*-PEG copolymers were prepared. The structure and molecule weight of the copolymers were characterized by 1H NMR (VarianUNITY INOVA400) and FT-IR (Perkin Elmer FT-IR spectrometer Frontier).

### 2.3. Fabrication and Characterization of Micelles with/without Drug

A DMSO (10 mL) solution of PAA-*g*-PEG graft copolymer (10 mg) was dropped into deionized water under vigorous ultrasonic agitation using a Type 60 Sonic Dismembrator (Fisher Scientific). The mixture was then dialyzed (Spectra/Por MWCO 8000–14000) against deionized water for 48 h to obtain the PAA-*g*-PEG micelle. For preparing MTX-incorporated micelle, PAA-*g*-PEG copolymer (10 mg) and MTX (1 mg) were dissolved in DMSO (5 mL) in a glass vial. The solution was then added dropwise to pure water (10 mL) under vigorous ultrasonic agitation. The resulting mixture was dialyzed against 1000 mL of deionized water for 24 h. The thus obtained micelle suspension was filtered through a 0.45 *μ*m membrane filter (Millipore) to remove the MTX aggregates. Subsequently, the mixture was ultrafiltered through a Millipore Centrifugal Filter Device (MWCO: 10,000) at 3500 r/min until the intraluminal fluid reached 3 mL to further remove unpacked free MTX and DMSO and concentrate the MTX-incorporated micelles suspension. The suspension was collected and freeze-dried to obtain MTX-incorporated micelles. The whole procedure was performed in dark.

Mean micelle diameters were measured on dynamic light scattering (DLS, Malvern Nano-ZS) using the micelle suspension (0.5 mg/mL) after filtered through a Durapore 0.22 *μ*m membrane (Millipore). The morphology of the micelles was analyzed using transmission electronic microscopy (TEM, Hitachi H-600). The micelle suspension sample (0.1 wt%) was dropped on a carbon-coated copper grid, followed by drying in air, and negatively staining with 3 wt% ammonium molybdate aqueous solution before observation. MTX dispersion in the micelles was analyzed by X-ray diffraction (XRD, Tongda TD-3500 diffractometer, Dandong, China) scanning from 5 to 40° with 0.06°/sec. The critical micelle concentration (CMC) was determined by fluorescence spectrophotometer (F-7000 fluorescence spectrophotometer, PerkinElmer) using pyrene as the fluorescence probe [35]. The drug loading content (DLC) and drug loading efficiency (DLE) was determined by ultraviolet spectroscopy measurement (PerkinElmer Lambda 650 S, excitation at 303 nm [37]) in DMSO using calibration curve obtained from MTX/DMSO solutions with different MTX concentrations and calculated as follows:

(1) DLC(wt%)=[weight  of  loaded  drugweight  of  drug  loaded  micelle]×100%,DLE(%)=[weight  of  loaded  drugweight  of  drug  in  feed]×100%.

### 2.4. *In Vitro* Drug Release Behaviors

*In vitro* drug release behavior of MTX-incorporated micelles was studied by a dialyzing method. Briefly, the suspensions of MTX-incorporated micelles in 2 mL of PBS buffer (10 mM, pH = 7.4) were dialyzed against 30 mL of PBS buffer (MWCO 8000–14,000) without DTT or containing 10 mM DTT to imitate the reducing environment. After predefined time intervals, 10 mL dialysate was replaced by equivalent fresh buffer. The amount of released MTX was calculated based on the absorbance intensity at 303 nm [37] using ultraviolet spectroscopy measurement using calibration curve obtained from MTX/PBS solutions with different MTX concentrations. Each batch sample was measured in triplicate.

### 2.5. Biocompatibility of PAA*-g*-PEG Micelles

Biocompatibility of the polymeric micelles was evaluated by coculturing the micelle suspensions with L929, HepG2, 4T1, and KB cells in 96-well plates (100 *μ*L, 4 × 10^3^ cells/well). The cells were preincubated for 24 h in DMEM (L929 and HepG2) or RPMI 1640 (4T1 and KB) supplemented with 10% fetal bovine serum, 1% benzylpenicillin/streptomycin at 37°C in a humidified atmosphere with 5% CO_2_. Then the culture media were replaced by corresponding medium (200 *μ*L) containing different concentration (0, 10, 40, 100, and 250 *μ*g/mL) of polymeric micelles. After incubated for another 48 h, the cell viability was determined by MTT assay [35].

### 2.6. Antiproliferative Activity of MTX-Loaded Micelles against Cancer Cell Lines

4T1, KB, and HepG2 cancer cell lines were seeded on 96-well plates (100 *μ*L, 4 × 10^3^ cells/well) and incubated for 24 h in DMEM (HepG2) or RPMI 1640 (4T1 and KB) supplemented with 10% fetal bovine serum, 1% benzylpenicillin/streptomycin at 37°C in a humidified atmosphere with 5% CO_2_. Then the culture medium was replaced with 200 uL of preprepared culture medium containing free MTX or MTX-incorporated micelles at different MTX concentration (0.01, 0.1, 0.5, 1.0, 5.0, 10.0, and 20.0 *μ*g/mL). The cells were cultured for another 48 h for 4T1 cells and 72 h for KB and HepG2 cells. Then, the cell viability was measured by MTT assay as described above.

## 3. Result and Discussion

### 3.1. Synthesis of the Reduction-Degradable PAA*-g*-PEG Copolymers

**Scheme 1:**
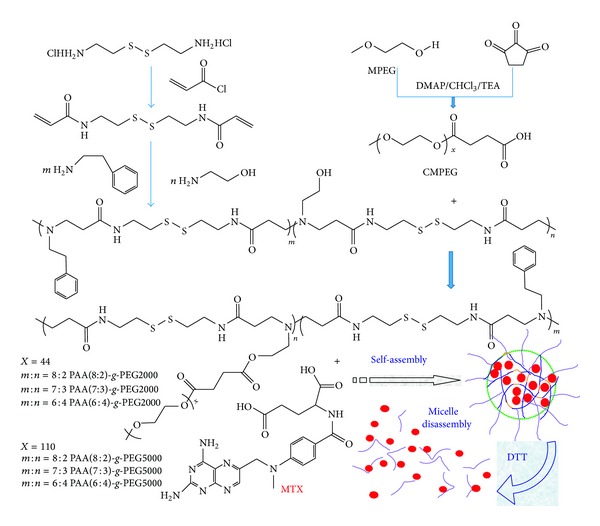
Chemical structure of reduction-degradable PAA-*g*-PEG amphiphilic copolymers and micelles.

6 kinds of reduction-degradable amphiphilic PAA*-g*-PEG copolymers were synthesized according to Scheme 1 as described in previous reports [35, 36]. At first, cystamine bisacrylamide was synthesized through a classical reaction involving the N-acylation of cystamine dihydrochloride by acryloyl chloride in a water/dichloromethane two-phase system. Next, the obtained cystamine bisacrylamide was reacted with primary amines by way of the Michael addition to form the polyamide amine (PAA). Finally, PAA-*g*-PEG copolymers were obtained by grafting MPEG-COOH onto the hydroxyl of PAA main chain using DCC as the condensing agent.

A mixture of phenylethylamine and ethanolamine were adopted as the amine compounds. The use of ethanolamine introduced hydroxyl into the polymer backbone for the condensation reaction in the next step. Phenylethylamine made the polymer backbone hydrophobic. Since interactions among the hydrophobic segments were the driving force in the formation of micelles, the interaction should strengthen the stability of micelles. The aromatic structure in phenylethylamine further provided potential benefit for stabilizing the drug-loaded micelles through the *π*-*π* stacking interaction with MTX. Changing the proportion of two amine compounds, three PAA(PAA(8:2), PAA(7:3), and PAA(6:4)) with different hydrophobic properties were obtained. These PAA copolymers were grafted with two kinds of MPEG-COOH (Mn = 2000/5000), respectively, to form 6 PAA-*g*-PEG graft copolymers (Table 1). As shown in Scheme 1, many disulfide (S-S) bonds were equably distributed throughout the backbone of the copolymers structure. These S-S bonds that came from cystamine were stable in normal condition and quickly fractured in reductive condition. Therefore, the micelles based on these amphiphilic copolymers were capable of keeping high stability in the absence of reductive agents, whereas quickly degrading under the reductive condition.

**Figure 1:**
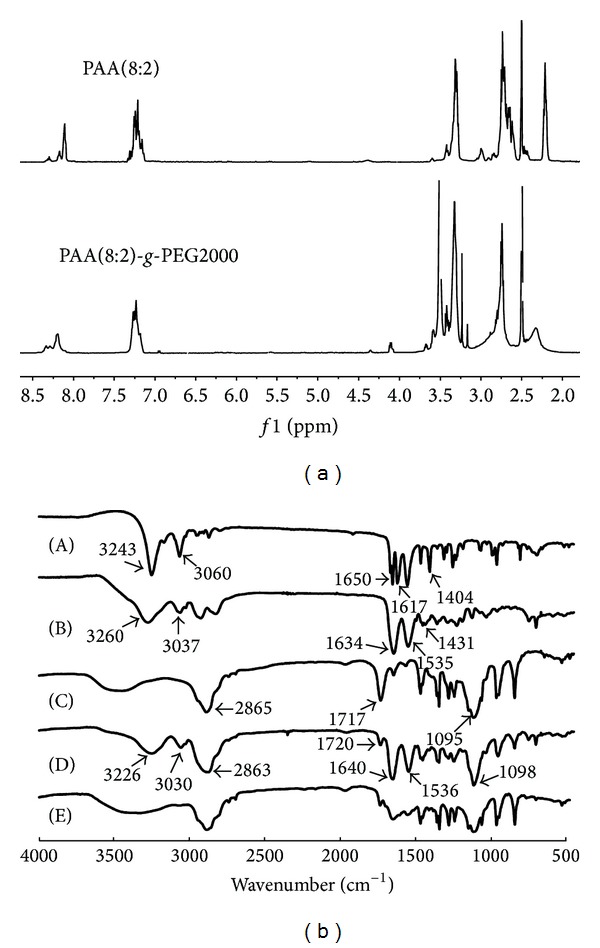
1H NMR spectra (a) of PAA(8:2) and PAA(8:2)*-g*-MPEG2000 and FTIR spectra (b) of cystamine bisacrylamide (A), PAA (b), MPEGCOOH (C), PAA(8:2)*-g*-MPEG2000 (D), and MTX-incorporated PAA(8:2)*-g*-MPEG2000 micelles (E).

The final structure of PEG-*g*-PAA was confirmed by ^1^H NMR (Figure 1(a)). The signals at 3.5 ppm (–CH_2_CH_2_–) indicated the presence of methylene group of the poly ethyleneglycol methyl ether methacrylate, and 4.15 ppm (–CH_2_CH_2_–O–CH_3_) indicated the presence of methyl group of the poly ethyleneglycol methyl ether methacrylate. Peak at 7.25 ppm (–C_6_H_5_–) indicated the presence of benzene of the phenylethylamine [38]. These ^1^H NMR results indicated that the structure of the copolymers was in agreement with the predicted structures as shown in Scheme 1.

In the FT-IR spectrum of PAA (Figure 1(b)-(B)), a new absorption at about 3010–3100 cm^−1^ typically for benzene appeared compared with cystamine bisacrylamide (Figure 1(b)-(A)). Meanwhile, in Figure 1(b)-(D), the ester peak at 1720 cm^−1^ almost completely disappeared, indicating the decrease of ester proportion in the copolymer in contrast with that in MPEGCOOH (Figure 1(b)-(C)). In addition, the PEG-*g*-PAA conjugate showed the intense stretching bands at 2863 cm^−1^ and 1098 cm^−1^ for PEG block, and a broad band –OC–NH– at about 3226 cm^−1^ and 3037, 1450 to 1640 cm^−1^. FT-IR spectra results indicated that the characteristic functional groups of the copolymers were in agreement with the predicted functional groups.

### 3.2. Fabrication and Characterization of the Micelles with/without MTX

**Figure 2:**
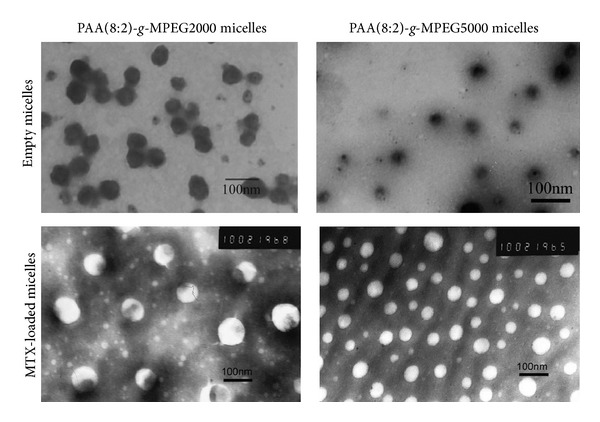
TEM images of PAA(8:2)*-g*-MPEG2000 micelle, PAA(8:2)*-g*-MPEG5000 micelle, MTX-incorporated PAA(8:2)*-g*-MPEG2000 micelle, and MTX-incorporated PAA(8:2)*-g*-MPEG5000 micelles.

MTX-incorporated micelles were fabricated by dialyzing PAA*-g*-PEG copolymer with MTX. After the incorporation of MTX, amide bond between 1450 and 1640 cm^−1^ merged to form a broad peak because of the C=N double bond in MTX (Figure 1(b)-(E)). The size, size distributions, and morphology of the micelles were analyzed by DLS and TEM. The representative DLS profiles and TEM images for MTX-incorporated micelles of copolymer PAA(8:2)-PEG2000 and PAA(8:2)-PEG5000 were shown in Figure 2, and results for all the 6 kinds of micelles were summarized in Table 2. Since TEM results were obtained under the condition of dehydration, which were a little different from the results obtained from DLS measurement in the aqueous solution. But these copolymers formed spherical blank micelles with diameter less than 70 nm. Generally, nanoparticles smaller than 100 nm were favorable for extravasation of the nanoparticles into tumors through EPR effect, because the discontinuous endothelium of tumor blood vessels form many pores ranging in size from 200 nm to 2 *μ*m with the average pore size approximately 400 nm on the vessel walls [39]. The micelles have relatively low CMCs, which was suitable for encapsulating hydrophobic drugs.

The theoretical drug loading content (DLC) was set at 9.09 wt%. The highest drug loading content and drug loading efficiency (DLE) were obtained in PAA(8:2)-PEG2000 micelles at 7.5% and 82%, respectively. With more hydrophobic segments, the DLC and DLE obviously increased, because more hydrophobic segments could increase the hydrophobic interaction of the copolymer hydrophobic segment and the hydrophobic interaction with the drug. Further, the introduction of phenyl group in the hydrophobic segment of the copolymer could provide *π*-*π* stacking between the drug and the copolymer, which could further increase the stability of the micelles. In our previous study, DLC of DOX-incorporated micelles with similar structure could reach 20% [36]. Compared to DOX, due to less aromatic structure and more amino and carboxyl in MTX, the maximum DLC for MTX was less than that for DOX. Both DLC and DLE decreased when increasing the density of PEG. The reason might be the decrease of the hydrophobic PAA in the core of the micelles. Considering the micelle size, DLC and DLE, PAA(8:2)*-g*-PEG2000 and PAA(8:2)*-g*-PEG5000 micelles were selected as typical examples for the further investigation.

**Figure 3:**
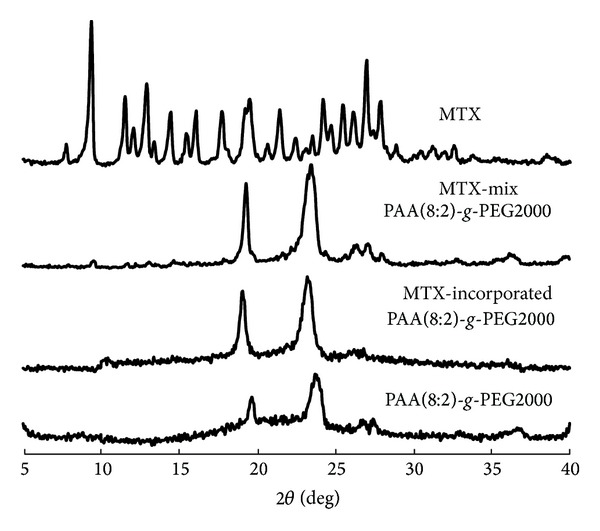
XRD diffractograms of MTX, MTX/PAA(8:2)*-g*-PEG2000 mixture, MTX-incorporated PAA(8:2)*-g*-PEG2000 micelles, and PAA(8:2)*-g*-PEG2000 micelles.

X-ray diffraction indicated that MTX dispersed uniformly inside the micelles. Free MTX was a crystalline compound as shown in Figure 3 for its characteristic diffraction pattern. PAA(8:2)*-g*-PEG2000 micelles without drug had two peaks at 2*θ* values of 19 and 23 in its diffractograms, probably due to the crystalline nature of the PEG segments. In the diffractograms of MTX and PAA(8:2)*-g*-PEG2000 mixture, MTX crystalline peaks at 2*θ* values of 10 and 27 could be detected. However, in the diffractograms of MTX-incorporated PAA(8:2)*-g*-PEG2000 micelles, nearly no other peak was found compared with that of the micelles without drug. The peaks at 2*θ* values of 19 and 23 did not change after the drug was incorporated, indicating that the crystalline structure of PEG layer was not disturbed. These results revealed that the MTX dispersed at the molecular level within the hydrophobic core of micelles.

### 3.3. Reduction-Triggered Drug Release* In Vitro*

**Figure 4:**
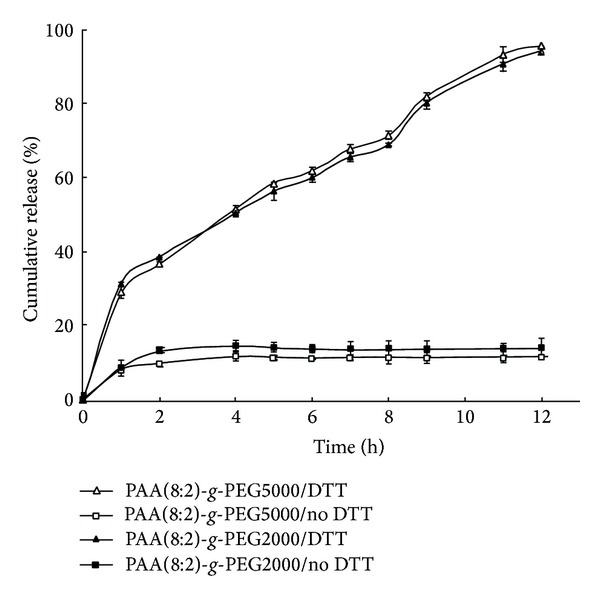
*In vitro* MTX release profiles of MTX-incorporated PAA(8:2)*-g*-PEG2000 and PAA(8:2)*-g*-PEG5000 micelles in PBS buffer solution (pH 7.4, 10 mM) at 37°C without DTT or containing 10 mM DTT.

The* in vitro* drug release behaviors of PAA-*g*-PEG micelles were carried out by dialysis against PBS buffered solution (pH 7.4, 10 mM) without DTT or containing 10 mM DTT, which was used as reductant to imitate the reducing environment in cancer cells. The MTX-release profiles of PAA(8:2)*-g*-PEG2000 and PAA(8:2)*-g*-PEG5000 in the absence and presence of DTT were shown in Figure 4. For an ideal nanoparticle drug delivery system, the drug release should be as less as possible before the nanoparticles arrived at the targeting cancer cells. However, small amount of drugs were unavoidably released in the neutral pH without the addition of reductive agent, which mimetic the physiological condition during the transfer process of micelles to the cancer cells. In the pH 7.4 PBS buffer solution without the existence of DTT, small amount of drug (about 10%) was released within the first 2 h. Thereafter, no tendency of further release was observed until 12 h. In a research of doxorubicin (DOX) encapsulated PEG-SS-PCL micelles, Zhong and coworkers reported that less than 20% of drug was released at neutral pH in 24 h [40]. The similar phenomenon was found in our prior reported work about DOX encapsulated PEG*-g*-PAA micelles [35]. Since MTX was an aromatic compound that was almost water insoluble, its hydrophobic nature and aromatic structure benefited its tight incorporation with the hydrophobic segment of the polymeric micelles and *π*-*π* stacking with the phenyl of PAA in the micelle core. Therefore, the initial fast release within 2 h was ascribed to small amount of drugs adhered on micelle PEG shell. After these loosely adhered drugs were washed off, the tightly encapsulated MTX in the micelle core was hardly released in PBS buffer without reductant. This property was in favor of keeping most of the drug in the micelles and delivering to the tumor site.

Remarkably, the PAA-*g*-PEG micelles released MTX rapidly in the presence of 10 mM DTT, a reductive environment analogous to that of the intracellular compartments such as cytosol in the cancer cells. As it can be found in Figure 4, there was a relatively faster release at the first 1 hour. Then, the accumulative release of MTX raised gradually to near 100% in 12 h. Obviously, the acceleration of MTX release in the present of DTT was resulted from the breakage of the micelle core due to the reduction-triggered cleavage of S–S bonds of the copolymer backbone [38]. It implied that the hydrophobic MTX was mainly entrapped in the hydrophobic cores of the micelles. As described above, the S-S bonds in the copolymer structure could be quickly reductively cleaved. Since the cleavage of the S–S bond in the hydrophobic segment would result in the lack of enough hydrophobic interaction of the core, the micelles could subsequently disassemble. The MTX incorporated in the core of the micelles then released along with the breakage of the micelle carriers.

This reduction-triggered breakage of micelles provided a useful releasing mechanism in cancer therapy in reducing side effect and enhancing tumor targeting. Because most MTX was incorporated in micelles core, the PEG shells might serve as a protective biological layer to improve micelle stability at normal physiological conditions (pH 7.4, without reductant) in preventing protein adsorption, elongating the circulation half-life in the blood stream, thus increasing the probability for tumor targeting through EPR effect. Once the micelles reached the tumor site, they might be internalized by cancer cells. The reductive intracellular environment might quickly break the S–S bond, resulting in the breakage of micelles and the fast release of the drug. This could be crucial for realizing the full therapeutic effect of MTX. Because the micelles were generally internalized by cells through the endocytotic pathway, MTX delivered by these nanoparticulate carriers were restricted within the endosome or lysosome [41–43]. The quick release of the MTX from the carriers thus provided the possibility for efficient endosome escape and enhanced its therapeutic effect. Therefore, this reduction-sensitive micelle might provide a promising approach for tumor targeting delivery of MTX.

### 3.4. Antiproliferative Activity of MTX-Incorporated Micelles on Cancer Cell Lines

**Figure 5:**
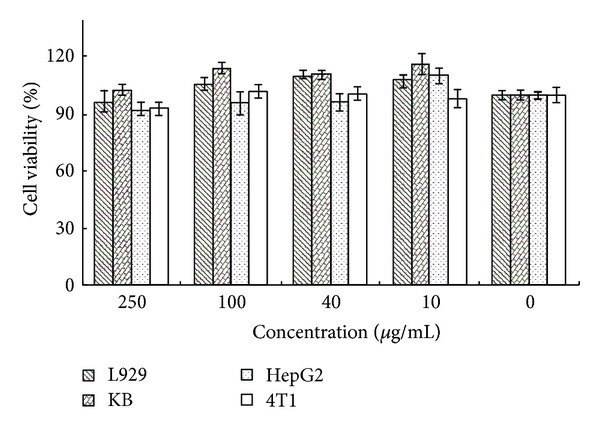
Biocompatibility assay of PAA(8:2)*-g*-PEG2000 micelles against L929, KB, HepG2, and 4T1 cells after incubation for 2 days. The standard deviation for each data point was averaged over five samples (*n* = 5).

The cytotoxicity of PEG-*g*-PAA micelles (without incorporation of MTX) was evaluated by coculturing different concentration of PAA(8:2)*-g*-PEG2000 micelles with L929 normal cells and HepG2, KB, 4T1 cancer cells. As shown in Figure 5, at low concentration (10 *μ*g/mL), all the cells proliferated well, with exceeded 100% cell viability compared with the control group which was cultured without the addition of micelles. Increasing the micelle concentration resulted in little decrease of the cell viability. But the cells still proliferated well. Even at very high micelle concentration (250 *μ*g/mL), all the cell viability could keep higher than 90%. These results indicated that the micelles did not show cytotoxicity against L929 normal cells and HepG2, KB, 4T1 cancer cells. Therefore, the copolymers were considered to have good biocompatibility.

**Figure 6:**
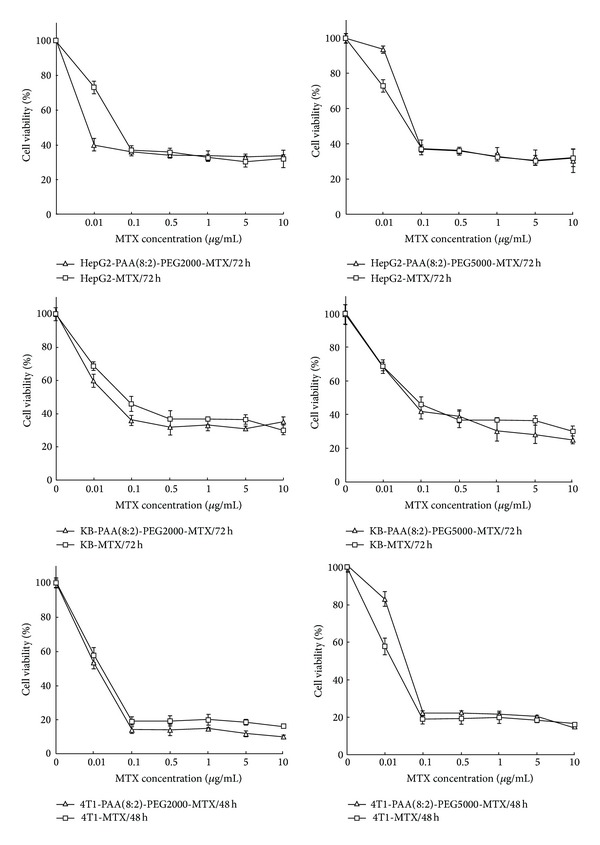
Antiproliferative activity of MTX-incorporated PAA(8:2)*-g*-PEG2000 micelles, PAA(8:2)*-g*-PEG5000 micelles, and free MTX on 4T1 after incubation for 2 days and KB and HepG2 cells after incubation for 3 days. The standard deviation for each data point was averaged over five samples (*n* = 5).

The antiproliferative activities of the MTX-incorporated micelles on different cancer cell lines were evaluated by examining the cell viability using MTT assay after being cocultured with MTX-incorporated micelles or free MTX. In order to investigate the effects of micelles on different tumor cells, 4T1, KB, and HepG2 cancer cell lines were chosen for the cellular growth inhibition test. Because the life cycle of 4T1 cell is different from that of KB and HepG2 cancer cells, studies on HepG2 and KB cancer cells were over 72 h and on 4T1 cancer cell was over 48 h. The final MTX concentrations in the culture medium were adjusted varying from 0.01 to 20 *μ*g/mL, and the results of MTT assay were shown in Figure 6. Both MTX-incorporated micelles and free MTX were observed dose-dependent antiproliferative activities against the three cancer cell lines. Since the MTX was a cytotoxic anticancer drug, these antiproliferative activities obviously came from the cytotoxicity of MTX, because the micelles without the incorporation of MTX did not affect the growth of cells. The MTX-incorporated micelles and free MTX showed similar inhabitation effect on these cancer cells, with a little slightly higher cytotoxicity of the PAA(8:2)*-g*-PEG2000 MTX-incorporated micelles than that of MTX itself, and a little slightly lower cytotoxicity of the PAA(8:2)*-g*-PEG5000 MTX-incorporated micelles.

Interestingly, at low MTX concentration (<0.1 *μ*g/mL), drug-loaded PAA(8:2)*-g*-PEG2000 micelles showed obviously stronger effect on killing cancer cells than pure MTX. But, with the increase of MTX concentration, the cytotoxicity of both the MTX-incorporated micelles and free MTX became nearly the same. The reason of these results might be due to the fact that, in the case of low MTX concentration, the internalization by the cancer cells of free MTX through diffusion mechanism might not be efficient because of the lack of driving force; namely, the difference of MTX concentration with intracellular and extracellular was low. When the MTX-incorporated PAA(8:2)*-g*-PEG2000 micelles were applied on cancer cells, the increase of concentration of the total formulation might enhance the intracellular uptake of MTX and improve proliferation inhibition efficiency at low MTX concentration. In contrast, for the PAA(8:2)*-g*-PEG5000 micelles, since MPEG was exposed on the micelle surface in aqueous solution, the thick hydrophilic PEG layer might delay the uptake of micelles into the cells and more micelles might remain in the media. This fact might be one of the reasons for the slightly lower cytotoxicity of PAA(8:2)*-g*-PEG5000 MTX-incorporated micelles [44].

When the MTX concentration exceeded 0.5 *μ*g/mL, the cytotoxicity of both micelles and the free MTX became very close, indicating that the MTX-incorporated micelles possessed comparable antiproliferative activities. It is a common phenomenon that the anticancer drugs delivery by nanoparticulate systems showed lower cytotoxicity than the drugs themselves, due to the entrapment of the nanoparticles within the endosome or lysosome and the delayed liberation of drugs from the carriers. Therefore, many stimuli-sensitive carrier systems were investigated to improving the delivery performance by accelerating the drug release after the carriers reached the cancer cells. The reduction-triggered breakable micelles in this present research could enter the cells through an endocytic mechanism, release the drug quickly inside the cells, and deliver the drug to the acting site in cytosol or inside membrane-bound cellular organelles. Therefore, these MTX-incorporated micelles that were able to realize rapid intracellular drug release might provide an effective tool for promoting the growth inhibition effect on cancer cells, especially within the low drug concentration scope.

## 4. Conclusion

Reduction-triggered breakable polymeric micelles incorporated with MTX were prepared using reductively breakable amphiphilic PAA-*g*-PEG copolymers. The micelles are spherical with diameters less than 70 nm. The shell-core structure of the micelles provided the properties to encapsulate the hydrophobic anticancer drug MTX in the hydrophobic core with DLC ranging from 2.9 to 7.5% and DLE 31.9 to 82.5%, which were highly dependent on the copolymer chemical structure. These micelles were capable of keeping stable and hold most MTX in the core in normal circumstance, whereas, in reductive environments that mimicked the intracellular compartments in the cancer cells, the entire drug payloads could be quickly released due to the reduction-triggered breakage of the micelles. Their antiproliferative activities on cancer cells were similar or slightly higher comparing with free MTX, especially within the low drug concentration scope.

## Figures and Tables

**Table 1:** Synthesis of amphiphilic polyamide amine-*g*-polyethylene glycol graft copolymers.

Entry	PAA		PAA-*g*-MPEG
	BAC/phenylethylamine /ethanolamine		Graft efficiency*
	Feed ratio	Measured ratio*	
PAA(8:2)-PEG2000	9/8/2	9/8/2	93%
PAA(7:3)-PEG2000	9/7/3	9/7/3	84%
PAA(6:4)-PEG2000	9/6/4	9/6/4	71%
PAA(8:2)-PEG5000	9/8/2	9/8/2	79%
PAA(7:3)-PEG5000	9/7/3	9/7/3	67%
PAA(6:4)-PEG5000	9/6/4	9/6/4	58%

*Calculated from peak areas of respective protons in ^1^H NMR.

**Table 2:** Properties of the reduction-triggered breakable micelles.

Series	Micelle size (nm)^a^		CMC (mg/L)^b^	DLC (%)^c^	DLE (%)^c^
	Without drug	Drug loaded			
PAA(8:2)-PEG2000	55	73	4.5	7.5	82.5
PAA(7:3)-PEG2000	37	56	20.1	6.3	69.3
PAA(6:4)-PEG2000	18	38	52.3	5.5	60.5
PAA(8:2)-PEG5000	34	42	9.4	6.9	75.9
PAA(7:3)-PEG5000	38	61	27.2	5.1	56.1
PAA(6:4)-PEG5000	70	98	44.0	2.9	31.9

^a^Micelle sizes were measured by DLS.

^b^Measured using pyrene as a fluorescence probe.

^c^Measured by fluorescence measurement.
